# Effect of Pre-Freezing 18 °C Holding Time on Post-Thaw Motility and Morphometry of Cryopreserved Boar Epididymal Sperm

**DOI:** 10.3390/ani15121691

**Published:** 2025-06-07

**Authors:** Mamonene Angelinah Thema, Ntuthuko Raphael Mkhize, Maleke Dimpho Sebopela, Mahlatsana Ramaesela Ledwaba, Masindi Lottus Mphaphathi

**Affiliations:** 1 Germplasm Conservation & Reproductive Biotechnologies, Agricultural Research Council, Animal Production, Private Bag X2, Pretoria 0062, South Africa; mamonenethema@gmail.com (M.A.T.); mrledwaba5@gmail.com (M.R.L.); 2 School of Agricultural, Earth and Environmental Sciences, University of KwaZulu-Natal, Private Bag X01, Pietermaritzburg 3209, South Africa; mkhizen31@ukzn.ac.za (N.R.M.); sebopela08@gmail.com (M.D.S.)

**Keywords:** boars, epididymis, cryopreservation, sperm motility, morphometry pre-freeze, post-thaw

## Abstract

The study investigated the sperm motility and morphometry of pre-freeze and post-thaw boar epididymal semen cooled at increasing holding times at 18 °C. A total of 50 testes of heterogeneous boars were collected (5 testes/day) from the local abattoir and transported to the laboratory at 5 °C within 30 min after slaughter. Semen was retrieved from the caudal part of the epididymis using the slicing float-up method, diluted with Beltsville Thawing Solution extender, pooled in a 50 mL centrifuge tube/5 testes/day, and cooled at 18 °C. Following each holding time (0, 3, 6, 9, 12, 24, and 48 h), the cooled semen sample was re-suspended with Fraction A extender and stored at 5 °C for an additional 45 min. A cooled resuspended semen sample was then diluted with Fraction B extender, loaded into 0.25 mL straws, and frozen using liquid nitrogen vapour. Thawing was accomplished by immersing the semen straws in warm (37 °C) water for 1 min and the samples were evaluated for sperm motility and morphometry traits using the computer-assisted sperm analyzer system. The data were analyzed using variance analysis. Descriptive statistics were used to assess sperm morphometry, establishing the minimum and maximum values. Boar epididymal sperm survived for up to 48 h when held at 18 °C. Furthermore, the highest post-thawed sperm motility rates were observed in semen frozen after 3 h of holding time, with a sperm total motility of 85.9%, a progressive motility of 60.3%, and a rapid motility of 33.2%, as compared to other holding times (*p* < 0.05). The acceptable ranges for pre-freeze and post-thawed sperm morphology were head length (8.4–9.1 µm), width (4.4–4.8 µm), area (29.9–38.2 µm^2^), perimeter (20.1–23.7 µm), midpiece width (1.1–2.8 µm), and sperm shape, were consistent regardless of the holding time. A holding time of 3 h enhances the cryoresistance of sperm cooled at 18 °C. Therefore, these findings suggest that boar epididymal sperm can be effectively conserved and can maintain fertilization capability when cooled for 3 h at 18 °C before freezing.

## Simple Summary

Semen cryopreservation enables the long-term storage and transportation of gametes that can be utilized for in vitro/vivo fertilization procedures. The cryopreservation of epididymal semen may be the only method to protect valuable male genetic material in the event of unanticipated death when ejaculation is no longer possible, and orchiectomy is required for an individual’s health. Approximately 40–50% of the boar sperm population does not survive the cryopreservation process. Freezing and thawing procedures subject boar sperm to extreme survival conditions, leading to structural and metabolic cell damage, sperm motility loss, and membrane integrity damage. Boar sperm is susceptible to cold shock due to a high content of polyunsaturated fatty acids in its plasm membrane. Several critical steps have been found to address the challenges faced when cryopreserving boar sperm. The pre-freeze stage, which involves maintaining the semen sample at 15–18 °C, is one noteworthy method. The goal of this procedure is to lessen the susceptibility of boar sperm to cold shock and preserve sperm viability. The prolonged holding time causes sperm capacitation processes to reverse and increases sperm cryoresistance. This study aims to investigate the sperm motility and morphometry of pre-freeze and post-thaw boar epididymal semen cooled at increasing holding times at 18 °C.

## 1. Introduction

The cryopreservation of epididymal semen might be the only method to protect valuable male genetic material in the event of unanticipated death [1]. The cryopreservation of boar epididymal semen may assist in conserving the genetic diversity of the threatened or endangered, making it easier for genetic material to be transported and exchanged over long distances [2,3]. The cryopreservation technique is frequently employed in reproductive biology labs to make the process of artificial insemination and in vitro fertilization easier and serves as a backup when infectious diseases arise in farms [4]. However, the use of post-thawed sperm is limited in pigs due to the high susceptibility of boar sperm to cold shock, caused by a high content of polyunsaturated fatty acids in its plasma membrane [5]. As a result, the cryopreservation of boar sperm may cause sperm damage, oxidative stress, thermal stress, protein denaturation, shrinkage, and irreversible membrane collapse [6]. Therefore, due to the intolerance of boar sperm to low temperatures, studies have reported improved results when extended ejaculated semen are cooled to 15–20 °C for a minimum of 1–3 h prior to freezing [7]. Typically, the initial step for boar semen cryopreservation protocols involves a 3 h holding time at 15–20 °C [8,9].

Diluted boar semen is commonly transported to processing laboratories in a liquid form at an optimum temperature of 15–20 °C [5]. To accommodate the transportation of semen, studies have performed tests on the variation in the sperm quality of other species for up to 24 h of holding time, and the cryotolerance has been recorded [10,11]. A holding time prior to freezing has proven to have a less discernible effect on the sperm. It has been proven to minimize the sensitivity of the sperm against oxidative stress that may be induced during cryopreservation [12]. Yeste et al. [9] described that holding time plays a major role in increasing the cryotolerance of sperm functionality. Santana et al. [13] described that holding time tends to reduce the metabolic activity of the sperm and its ability to preserve its viability. In addition, Torres et al. [14] recorded holding time as having improved the cryopreservation of ejaculated boar semen and allowed prolonged interaction between the sperm and the seminal plasma components.

The major disadvantage of using post-thaw boar semen is the functional alterations to the sperm, such as its morphometry and motility traits, which may be compromised during freezing. It has been proven that sperm with normal motility, but abnormal morphologies are incapable of fertilization [15]. Sperm morphometry has been associated with factors that can determine fertility [16]. The study by García-Herreros et al. [17,18] reported smaller boar sperm heads following the cryopreservation of the ejaculated semen. Due to technical discrepancies, subjective sperm morphology assessment in pigs remains troublesome [19]. Therefore, to lessen the subjective way of analyzing sperm morphometry, the computer-assisted sperm analyzer (CASA) system approach was developed and established [20]. The accurate, precise, repeatable, and reliable results have been reported by a few studies when using the CASA system, which had been a challenge for the subjective analyzer [21,22].

Information about the effects of holding time on post-thaw boar epididymal sperm survival has received less attention. To our knowledge, no published study has documented boar epididymal sperm morphometry and motility induced by holding time at pre-freeze and post-thaw. Therefore, investigating the epididymal sperm motility and morphometry of pre-freeze and post-thaw semen will assist in determining the fertilization and cryopreservation capability of epididymal sperm in pigs. This study aims to investigate the variability in the sperm motility and morphometry of pre-freeze and post-thaw semen cooled to 18 °C for prolonged holding times.

## 2. Materials and Methods

### 2.1. Boar Epididymal Semen Retrieval

A total of 50 testes of heterogeneous boars were collected (5 testes/day) from the local abattoir and transported to the laboratory at 5 °C within 30 min after slaughter. The testes were further stored at 5 °C for an additional 2 h before being processed. Following storage, the caudal epididymides and the proximal ducts were removed from the testes and cleaned with Sabax^®^ saline NaCl 0.9%, solution (Adcock Ingram, Johannesburg, South Africa). To avoid blood contamination, superficial blood vessels were punctured so that most of the blood could be wiped off. Then, semen samples were retrieved from the cauda epididymides using the slicing float-up method. The slicing float-up method was performed by slicing the cauda epididymides with a blade to relieve the semen into the Petri dish (Whitehead Scientific, Cape Town, South Africa) and this was diluted with 5 mL of Beltsville Thawing Solution (BTS; IMV Technologies, L’Aigle, France), a semen extender.

### 2.2. Boar Epididymal Semen Dilution

The retrieved semen samples of good quality (>80% total sperm motility) from 5 cauda epididymis/day were pooled and transferred into 50 mL centrifuge tubes (Whitehead Scientific, Cape Town, South Africa). The semen samples were diluted at a ratio of 1:4 (*v*/*v*) with a BTS extender. The diluted semen samples were cooled to 18 °C and cryopreserved for different holding times (0, 3, 6, 9, 12, 24, and 48 h).

### 2.3. Preparation of Boar Epididymal Semen Freezing Extender

The semen holding and freezing extender was prepared one day before use. The boar semen extender used for holding was the BTS extender, which was prepared by dissolving a sachet of BTS extender in a litre of Sabax^®^ (Adcock Ingram, Johannesburg, South Africa) distilled water and stored at 18 °C. The chemical composition of the BTS extender is presented in Table 1. The semen freezing extender was separated into fractions A and B and stored at 5 °C. Both Fraction A and Fraction B were prepared according to Thema et al. [23]. The Fraction A extender consisted of BTS + 15% egg yolk, and the Fraction B extender consisted of BTS + 15% egg yolk + 3% Glycerol: *v*/*v*.

### 2.4. Boar Epididymal Semen Cryopreservation and Thawing Process

Diluted semen with a BTS extender was cooled to 18 °C and subjected to freezing for different holding times (0, 3, 6, 9, 12, 24, and 48 h). At the end of each holding time, the semen was then re-suspended with Fraction A extender and cooled to 5 °C for an additional 45 min. Furthermore, the cooled semen was then diluted with a Fraction B extender [23] and loaded into 0.25 mL straws (Embryo Plus, Brits, South Africa). The semen straws were exposed to 3 cm above liquid nitrogen for 20 min during cryopreservation [24]. The frozen semen straws were immediately transferred into the liquid nitrogen tank (−196 °C) for storage. Thawing was accomplished by removing the frozen straws from the liquid nitrogen tank, exposing them to air for 5 s, and immersing the semen straws in warm (37 °C) water for 1 min.

### 2.5. Evaluations of Boar Epididymal Sperm Motility Traits

Sperm motility traits were analyzed following each holding time (0, 3, 6, 9, 12, 24, and 48 h) at pre-freeze and post thaw using the CASA system (Sperm Class Analyzer^®^ 6.6.80, Microptic S.L., Barcelona, Spain) with different slides. The volume of the 5 µL semen sample at pre-freeze or post-thaw following each holding time was transferred to a warmed (37 °C) microscope glass slide (Labchem Pty Ltd., Cape Town, South Africa) and covered with a warm coverslip (Labchem Pty Ltd., Cape Town, South Africa). Two fields of pre-freeze and post-thaw semen following each holding time with an average of 250 sperm (34.3 × 10^6^/mL/replication) were captured with the aid of a microscope (Nikon^®^, Toyko, Japan) at a 10× magnification using the CASA system.

### 2.6. Semen Staining Preparation for Boar Epididymal Sperm Morphometry

Semen samples were stained following each holding time (0, 3, 6, 9, 12, 24, and 48 h) at pre-freeze and post thaw. Prior to staining, the semen sample at pre-freeze or post-thaw stage following each holding time was diluted at a ratio of 1:10 with the BTS extender. The diluted sample was further fixed with 4% Formaldehyde for 10 min. Then, 10 µL of the fixed sample at pre-freeze or post thaw following each holding time was smeared on the microscope glass slide and allowed to air-dry overnight at room temperature. During staining, the dried microscope glass slide sample was gently dipped into the SpermBlue^®^ (Ducit, Centurion, South Africa) tray for 1 min and into distilled water for 5 s (to remove debris and ensure a clean background). A paper towel was used to wipe the back of the microscope glass slide and allowed air-dry at a room temperature. Following drying, the slide was mounted using the Dibutylphthalate Polystyrene Xylene mounting medium (Inqaba Biotechnical Industries (Pty) Ltd., Pretoria, South Africa) and covered with the coverslip.

### 2.7. Analysis of Boar Epididymal Sperm Morphometry

Boar epididymal sperm morphometric traits were evaluated using the CASA system. At least 100 sperm were measured following each holding time for 10 replications (days of collection) (100 × 10 = 1000) of pre-freeze and post-thaw semen. Overall, 7000 pre-freeze (0, 3, 6, 9, 12, 12, 24, and 48 h) sperm and 7000 post-thaw (0, 3, 6, 9, 12, 24, and 48 h) sperm were analyzed for morphometry traits. The microscope glass slide was observed (the focus knob was used to focus on the sperm) and measured for the sperm head area (A; µm^2^), head perimeter (P; µm), head length (L; µm), head width (W; µm), head ellipticity (length/width), head elongation (L − W/L + W), head regularity (π × (L.W/4.A), head roughness [4 π × (A/P^2^)], midpiece width, and shape indices (sperm with a normal head, acrosome, and midpiece, or an abnormal head shape—amorphous, pyriform, or tapered).

### 2.8. Statistical Analysis

The sperm morphometric data were divided into two groups: The first group were the measurements of primary sperm head traits (length, L; area, A; width, W; perimeter, P; ellipticity, L/W; rugosity, 4 πA/P2; elongation, L − W/L + W; and regularity, πL.W/4.A). The second group was the determination of the sperm head indices (head type, normal, shape, acrosome defect, and midpiece defect). The first sperm morphometric data group: In the first sperm morphometric data group, descriptive statistics were used to establish the minimum and maximum values (threshold) using 10–90 percentiles for every morphometric trait in this group.

The data for the first sperm morphometric data group and the sperm motility traits were analyzed using repeated-measure analysis of variance (ANOVA). The Shapiro–Wilk’s test was performed on the standardized residuals to test for deviations from normality. In cases where significant deviation from normality was observed and due to skewness, outliers were removed until this was normal or symmetrically distributed. Student’s t-LSDs (least significant differences) were calculated at a 5% significance level to compare means of the significant source effect of holding time on the first sperm morphometric data group and the motility traits at pre-freeze and post thaw. The ANOVA analysis was performed using SAS version 9.4 statistical software.

The second sperm morphometric data group was analyzed using a Chi-square test to determine the effect of holding time on the sperm percentage variations regarding the sperm with a normal head, acrosome, and midpiece, or an abnormal head shape (amorphous, pyriform, or tapered) evaluated at pre-freeze and post thaw. The scores were subjected to a 1:1 frequency table, and a Chi-square (χ^2^) test was performed to test for equal proportions. Contingency R × C frequency tables were performed for associations between holding times at pre-freeze and post thaw. The tests were performed for association (patterns) and, where significant differences were found, graphs were constructed to demonstrate the differences in these patterns. The Chi-square analysis was performed using SAS version 9.4 statistical software.

## 3. Results

**Figure 1:**
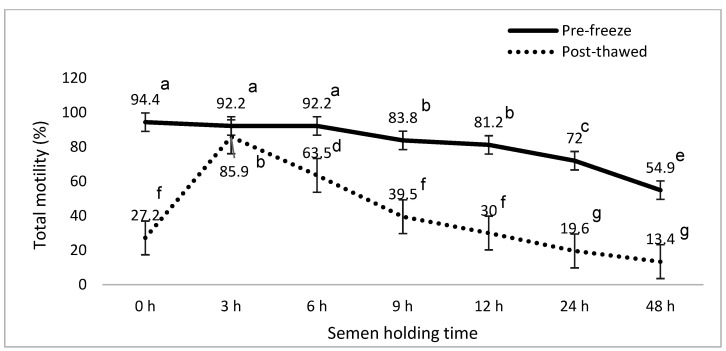
The effect of boar epididymal semen holding time on pre-freeze and post-thaw sperm total motility traits using CASA system (mean ± SD). ^a–g^ Different letters indicate the significant difference between the pre-freeze and post-thaw groups at specific holding times (*p* < 0.05).

**Figure 2:**
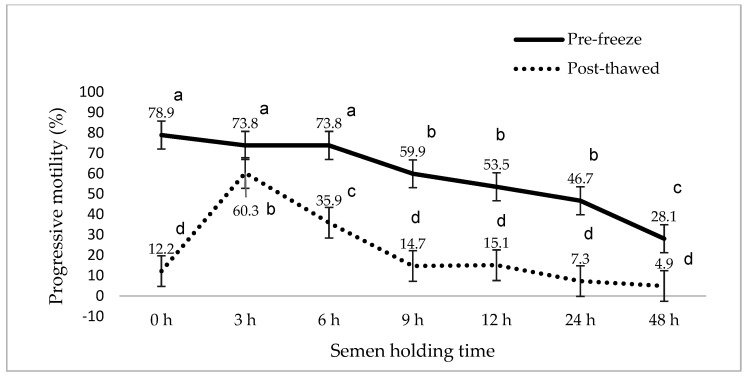
The effect of boar epididymal semen holding times on pre-freeze and post-thaw sperm progressive motility traits using the CASA system (mean ± SD). ^a–d^ Different letters indicate the significant difference between the pre-freeze and post-thaw groups at specific holding times (*p* < 0.05).

**Figure 3:**
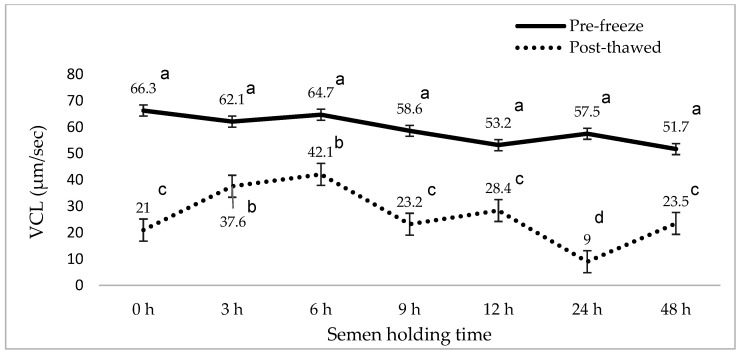
The effect of boar epididymal semen holding time on pre-freeze and post-thaw sperm curvilinear velocity traits using the CASA system (mean ± SD). ^a–d^ Different letters indicate the significant difference between the pre-freeze and post-thaw groups at specific holding times (*p* < 0.05). VCL = curvilinear velocity.

**Figure 4:**
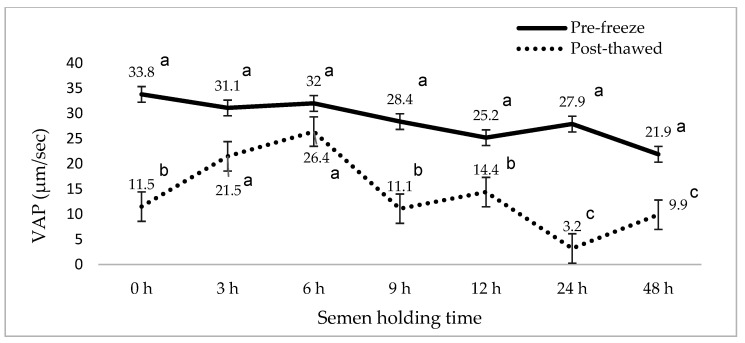
The effect of boar epididymal semen holding time on pre-freeze and post-thaw sperm average pathway velocity traits using the CASA system (mean ± SD). ^a–c^ Different letters indicate the significant difference between the pre-freeze and post-thaw groups at specific holding times (*p* < 0.05). VAP = average pathway velocity.

**Figure 5:**
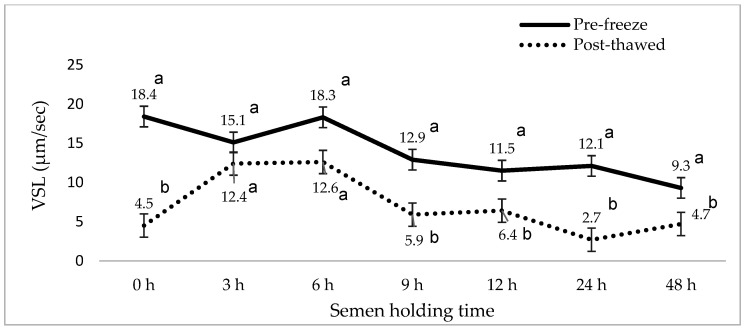
The effect of boar epididymal semen holding time on pre-freeze and post-thaw sperm straight line velocity traits using the CASA system (mean ± SD). ^a–b^ Different letters indicate the significant difference between the pre-freeze and post-thaw groups at specific holding times (*p* < 0.05). VSL= straight line velocity.

**Figure 6:**
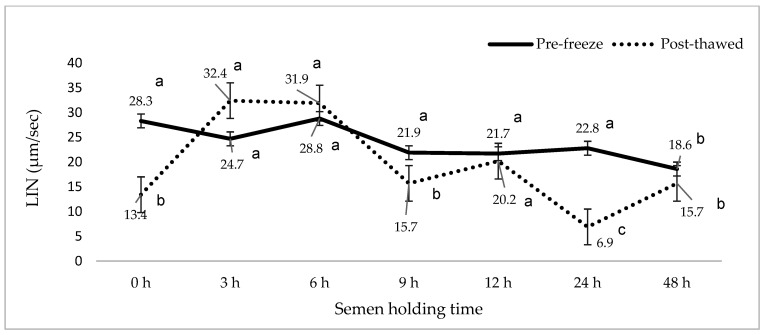
The effect of boar epididymal semen holding time on pre-freeze and post-thaw sperm linearity traits using the CASA system (mean ± SD). ^a–c^ Different letters indicate the significant difference between the pre-freeze and post-thaw groups at specific holding times (*p* < 0.05). LIN= linearity.

Figure 1, Figure 2, Figure 3, Figure 4, Figure 5 and Figure 6 represent the effect of boar epididymal semen holding time on pre-freeze and post-thawed sperm motility and velocity traits. Semen frozen immediately after retrieval (0 h) recorded decreased post-thaw sperm total (27.2 vs. 94.4%) and progressive (12.2 vs. 78.9%) motility versus the values recorded at the prolonged holding time at pre-freeze (*p* < 0.05). Contrary to expectations, this study find a significant difference between the pre-freeze and post-thaw sperm total (92.2%; 85.9%) motility recorded at the 3 h holding time. The highest post-thaw sperm progressive motility (60.3%) was recorded in the semen frozen after a 3 h holding time compared to the other holding times (*p* < 0.05). More than 70% of pre-freeze sperm progressive motility was recorded between the 0 and 6 h holding times and then this decreased during the 9–48 h prolonged holding times. The present study recorded boar epididymal semen as having survived for 48 h of holding time, with >50% sperm total motility recorded at pre-freeze. Furthermore, semen cooled for 3–48 h prior to freezing recorded significant post-thaw sperm VCL, VSL, and VAP movement. Furthermore, there was no significant difference between the post-thaw sperm velocity values recorded when the semen was cooled for 3–6 h prior to freezing.

**Figure 7:**
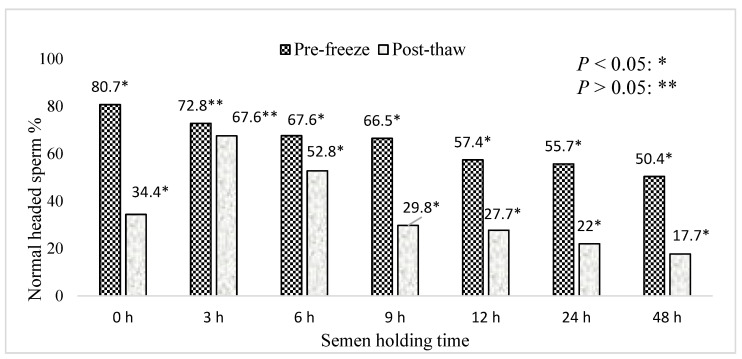
The effect of boar epididymal semen holding time on the percentage of pre-freeze and post-thaw sperm with a normal head. The significant difference (*p* < 0.05) is represented by a star and non significant difference (*p* > 0.05) is represented by two stars within the bars.

Figure 7 represents the effect of holding time on the percentage of pre-freeze and post-thaw boar epididymal sperm with normal heads. The present study recorded < 40% of post-thaw sperm with normal heads in the semen frozen immediately after retrieval (0 h). Furthermore, less than 30% of post-thaw sperm with a normal head were recorded in the semen samples frozen for 9–48 h compared to the 3 h holding time (*p* < 0.05). The percentage of pre-freeze sperm with a normal head decreased with the increasing holding time of the semen samples. Greater than 60% of the post-thaw sperm with a normal head were recorded in the semen samples frozen for a 3 h holding time, compared to the other holding times (*p* < 0.05). Moreover, a 6 h holding time maintained >50% of post-thaw sperm with normal heads.

**Figure 8:**
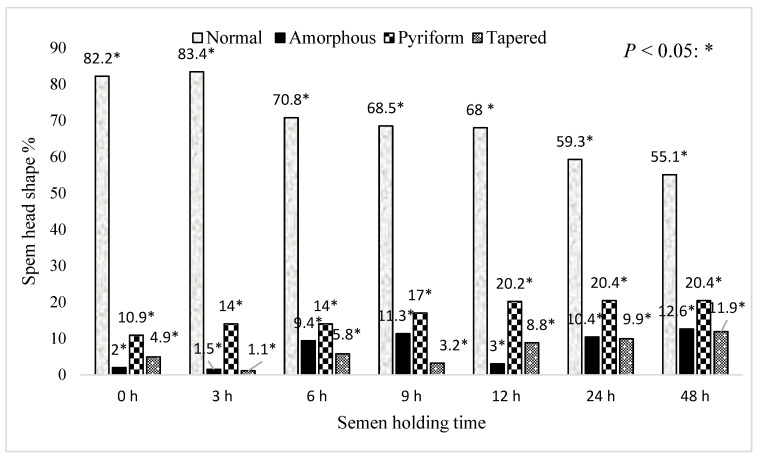
The effect of holding time for boar epididymal semen on the percentage of pre-freeze sperm with a normal head shape. The significant difference (*p* < 0.05) is represented by a star within the bars.

Figure 8 represents the effect of holding time for boar epididymal semen on the percentage of pre-freeze sperm with a normal head shape. Pre-freeze sperm with a normal head shape decreased with the increasing time of holding the semen samples (*p* < 0.05). Greater than 20% of pre-freeze sperm with a pyriform shape were recorded between 12 and 24 h of holding time. Whereas, only <20% of pre-freeze sperm with an amorphous or tapered shape were recorded for the 48 h holding time.

**Figure 9:**
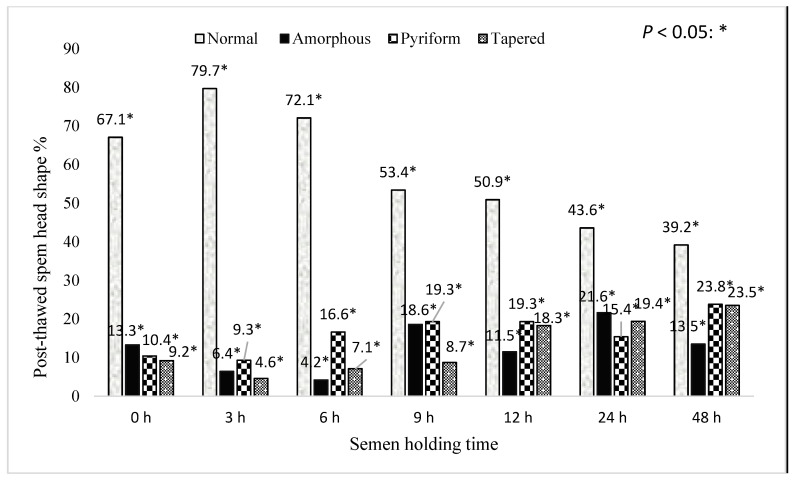
The effect of holding time for boar epididymal semen on the percentage of post-thaw sperm with a normal head shape. The significant difference (*p* < 0.05) is represented by a star within the bars.

Figure 9 represents the effect of holding time for boar epididymal semen on the percentage of post-thawed sperm with a normal head shape. The percentage of post-thawed sperm with a normal head shape decreased with increased semen sample holding time (*p* < 0.05). Greater than 70% of the post-thaw sperm with normal head shapes were recorded in the semen frozen for the 3 and 6 h holding times, as compared to the other holding times (*p* < 0.05). Furthermore, >21.6% of the post-thaw sperm with an amorphous shape were recorded in the semen frozen for the 24 h holding time. Meanwhile, an increase in post-thaw sperm with tapered (23.5%) and pyriform (23.5%) shapes was recorded in the semen samples frozen for the 48 h holding time.

**Figure 10:**
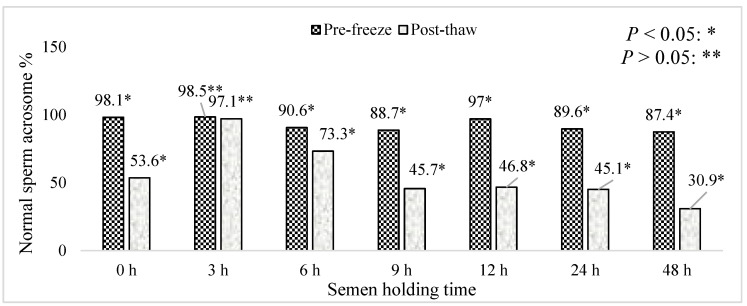
The effect of boar epididymal semen holding time on the percentage of pre-freeze and post-thaw sperm with a normal acrosome. The significant difference (*p* < 0.05) is represented by a star and the non significant difference (*p* > 0.05) is represented by two stars within the bars.

The effect of boar epididymal semen holding time on the percentage of pre-freeze and post-thaw sperm with a normal acrosome is represented in Figure 10. Greater than 80% of the pre-freeze sperm with a normal acrosome was recorded in the present results regardless of holding time. The percentage of post-thaw sperm with a normal acrosome decreased with the increasing holding time of the semen samples (*p* < 0.05). Semen samples frozen for the 9–48 h holding times recorded <50% of post-thaw sperm with a normal acrosome. Furthermore, semen frozen immediately after retrieval, recorded <60% of post-thaw sperm with a normal acrosome. Meanwhile, semen exposed to the 3–6 h holding times recorded >70% of post-thawed sperm with a normal acrosome.

**Figure 11:**
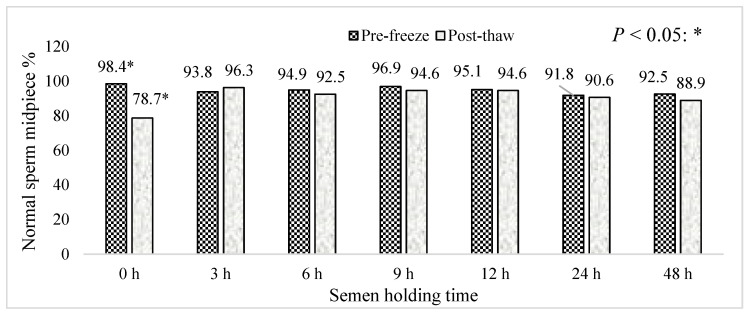
The effect of boar epididymal semen holding time on the percentage of pre-freeze and post-thaw normal sperm midpieces. The significant difference (*p* < 0.05) is represented by a star within the bars.

Figure 11 represents the effect of boar epididymal semen holding time on the percentage of pre-freeze and post-thaw normal sperm midpiece. Semen frozen immediately after retrieval recorded the lowest percentage of post-thaw normal sperm midpieces. Overall, >80% of post-thaw and pre-freeze sperm with normal midpieces were recorded throughout 48 h of holding time.

Table 2 represents the effect of boar epididymal semen holding time on pre-freeze sperm morphometric traits. The present study recorded acceptable pre-freeze sperm head length (8.7–9.1 µm), width (4.5–4.8 µm), area (33.3–36.6 µm^2^), perimeter (21.4–23.7 µm), and midpiece width (1.1–2.8 µm) which fell within the normal range. No significant difference was recorded between the elongation and regularity throughout the semen holding time.

The effect of boar epididymal semen holding time on post-thaw sperm morphometric head traits is presented in Table 3. The present study recorded the highest post-thaw sperm head length (9.1 µm) and area (38.2 µm) in the semen samples frozen for more than 3 h of holding time. Whereas semen frozen immediately after retrieval recorded the lowest post-thaw sperm head length (8.4 µm), area (4.4 µm^2^), and width (29.9 µm). Overall, semen frozen for 0–48 h of holding time recorded acceptable post-thaw sperm head length (8.4–9.1 µm), width (4.4–4.6 µm), area (29.9–38.2 µm^2^), perimeter (20.1–23.1 µm), and midpiece width (1.0–1.3 µm), which fell within the normal range (*p* < 0.05). No significant difference was recorded in post-thaw sperm midpiece elongation and regularity.

## 4. Discussion

This study set out with the aim of investigating the variability in the sperm motility and morphometry for pre-freeze and post-thaw semen cooled to 18 °C for prolonged holding times. The present study recorded boar epididymal pre-freeze and post-thaw sperm as having survived up to 48 h of holding time. However, semen frozen immediately after retrieval (0 h) and for prolonged holding times (9–48 h) recorded less cryoresistance with the lowest post-thawed sperm total and progressive motility percentages. A possible explanation for these results might be that semen retrieved from the epididymis contains epididymal fluid instead of seminal plasma [25], suggesting that holding time plays a significant role in minimizing the sensitivity of the sperm to cold shock through seminal plasma proteins [12].

One unanticipated finding was that >80% of post-thaw sperm total motility was recorded in the semen frozen for the 3 h holding time. This study produced results which corroborate the findings of a range of previous works in this field. These results match those observed in a previous study by Rath and Nieman [26], who also reported greater than 70% of post-thaw sperm retaining total motility in boar epididymal semen frozen for a 4 h holding time. This study also accords with the observations by Okazaki et al. [27], which recorded 80% of post-thaw sperm retaining total motility in boar epididymal semen compared to ejaculated semen. A possible explanation for these results might be that holding time protects the boar sperm against cold shock through the maintenance of the lipid architecture of the plasma membrane [7]. The observed increase in post-thawed sperm total and progressive motility recorded for the 3 h holding time could be attributed to the epididymal sperm in the testis mixing with the epididymal fluids and remaining quiescent until they are ejaculated in semen [28]. This suggests that the protein profile in the epididymal fluid could be responsible for the increase in boar epididymal sperm cryoresistant [29]. In addition, the cauda part of the epididymis consists of immobilin and antioxidants, which play a role in increasing epididymal fluid viscosity [30].

In accordance with the present results, previous studies demonstrate high cryotolerance in the sperm of boar epididymis compared to the ejaculated semen. This is supported by studies on cryopreservation of epididymal semen in humans [31] and equines [18,32], where it had recorded higher sperm motility rates following thawing. Moreover, these results are supported by our previous studies on the cryopreservation of Windsnyer [24] and large white boar semen [33], in which the ejaculated sperm was also demonstrated to have lower cryotolerance. Although, these results agree with the published studies on the cryopreservation of boar epididymal semen, they differ from those by Guthrie and Welch [10], which reported 43.4% of post-thaw sperm retaining total motility in ejaculated boar semen samples frozen for a 3 h holding time. Additionally, these results differ from Torres et al. [13] and Tomás et al. [27], which also reported a 24 h holding time to be ideal for the freezing of ejaculated boar semen. Although a 24 h holding time has been reported to be the best in studies on the cryopreservation of ejaculated semen, their results recorded the lowest post-thaw sperm motility rates irrespective of the holding time. These discrepancies between the cryotolerance of ejaculated and epididymal sperm may be related to the electrolytes in the seminal plasma of the ejaculated semen, which may have a negative effect on the sperm and lead to a decrease in its quality during cryopreservation in comparison with epididymal semen [18]. Seminal plasma may increase the risk of premature capacitation and membrane destabilization, which can compromise sperm cryoresistance [30].

A greater percentage of post-thaw sperm with normal heads and acrosomes were recorded in the semen frozen after 3 and 6 h of holding time. This study highlights that cryopreservation can have a greater impact on the sperm head shape and acrosome integrity, especially in the semen frozen immediately after retrieval and following increased holding time (9–48 h). The changes in the sperm head shape were associated with a prolonged holding time, cold shock, oxidative stress, and premature capacitation which may be responsible for the low fertility rate. This finding corroborates the ideas of [34] who suggested that the sperm plasma membrane is very sensitive and may break in the acrosomal area during cryopreservation. The present study differs from the study by García-Herreros et al. [17], which recorded the largest changes in the morphometric dimensions of post-thaw ejaculated boar sperm.

## 5. Conclusions

The present study recorded boar epididymal pre-freeze and post-thaw sperm as having survived up to 48 h of holding time. However, the pre-freeze holding time of the boar epididymal semen at 18 °C for 3–6 h resulted in significantly higher sperm total motility and morphometry. Additionally, the 3 and 6 h holding times were able to improve the cryotolerance of the boar epididymal sperm. The freezing of semen immediately after retrieval and for a prolonged holding time of 9–48 h negatively affected sperm quality, due to the absence of the seminal plasma in the epididymal semen. This suggests a protective role of seminal plasma in minimizing the oxidative stress of epididymal sperm frozen immediately after retrieval and for prolonged a holding time (9–48 h). This study concludes that even after the death of the boar, it may be possible to conserve valuable genetic material (epididymal sperm). Additionally, these results show that the study was successful as it was able to overcome the challenge of cryopreserving boar sperm. It was further observed that the boar epididymal sperm exhibits better cryotolerance compared to results for ejaculated sperm obtained in other studies. Therefore, further research should be carried out to investigate the fertilizing ability of pre-freeze and post-thaw epididymal boar sperm.

## Figures and Tables

**Table 1:** Chemical composition of Beltsville Thawing Solution extender.

Composition	Fraction A
**Glucose**	37.0
**Sodium citrate**	6.0
**Ethylene diaminuteetraacetic acid**	1.25
**Sodium bicarbonate**	1.25
**Gentamycin**	-
**Penicillin**	1.10
**Streptomycin**	1.10
**Potassium chloride**	0.75

**Table 2:** The effect of boar epididymal semen holding time on pre-freeze sperm morphometric head traits (mean ± SD).

	Holding time						
	0 h	3 h	6 h	9 h	12 h	24 h	48 h
**Pre-freeze sperm head trait**							
**Length (µm)**	9.1 ± 0.8 ^a^	9.0 ± 0.5 ^a^	8.9 ± 0.8 ^b^	8.8 ± 0.6 ^b^	8.9 ± 0.5 ^b^	8.9 ± 0.7 ^b^	8.7 ± 0.6 ^c^
**Width (µm)**	4.5 ± 0.5 ^b^	4.7 ± 0.3 ^a^	4.7 ± 0.4 ^a^	4.7 ± 0.3 ^a^	4.8 ± 0.3 ^a^	4.6 ± 0.4 ^b^	4.7 ± 0.3 ^a^
**Area (µm^2^)**	36.6 ± 5.7 ^a^	34.8 ± 3.2 ^b^	34.1 ± 4.0 ^b^	33.3 ± 3.3 ^c^	34.6 ± 2.9 ^b^	33.0 ± 3.9 ^c^	33.0 ± 3.6 ^c^
**Perimeter (µm)**	21.4 ± 2.6 ^b^	23.7 ± 1.2 ^a^	23.3 ± 1.8 ^a^	23.0 ± 1.3 ^a^	23.5 ± 1.1 ^a^	23.1 ± 1.7 ^a^	23.1 ± 1.3 ^a^
**Pre-freeze sperm midpiece trait**							
**Width (µm)**	1.1 ± 0.3 ^c^	2.8 ± 1.1 ^a^	2.7 ± 1.2 ^a^	2.2 ± 1.1 ^b^	2.6 ± 1.0 ^a^	2.4 ± 1.2 ^b^	2.6 ± 1.3 ^a^
**Pre-freeze sperm shape indices**							
**Ellipticity**	2.0 ± 0.4	1.9 ± 0.1	1.9 ± 0.1	1.9 ± 0.1	1.9 ± 0.1	1.9 ± 0.1	1.9 ± 0.1
**Elongation**	0.3 ± 0.1	0.3 ± 0.1	0.3 ± 0.1	0.3 ± 0.1	0.3 ± 0.1	0.3 ± 0.1	0.3 ± 0.1
**Regularity**	0.9 ± 0.0	0.9 ± 0.0	0.9 ± 0.0	0.9 ± 0.0	0.9 ± 0.0	0.9 ± 0.0	0.9 ± 0.0
**Roughness**	1.0 ± 0.3	0.8 ± 0.0	0.8 ± 0.0	0.8 ± 0.0	0.8 ± 0.0	0.8 ± 0.0	0.8 ± 0.0

^a–c^ Values with different superscripts differ significantly in the same row (*p* < 0.05).

**Table 3:** The effect of holding time on the post-thaw boar epididymal sperm morphometric head trait (mean ± SD).

	Holding time						
	0 h	3 h	6 h	9 h	12 h	24 h	48 h
**Post-thawed sperm head trait**							
**Length (µm)**	8.4 ± 0.7 ^c^	9.1 ± 0.9 ^a^	8.7 ± 0.7 ^b^	8.7 ± 0.8 ^b^	8.7 ± 0.4 ^b^	8.7 ± 0.6 ^b^	8.8 ± 0.9 ^b^
**Width (µm)**	4.4 ± 0.4 ^b^	4.5 ± 0.4 ^a^	4.6 ± 0.4 ^a^	4.6 ± 0.5 ^a^	4.6 ± 0.3 ^a^	4.5 ± 0.4 ^a^	4.5 ± 0.7 ^a^
**Area (µm^2^)**	29.9 ± 3.3 ^c^	38.2 ± 6.9 ^a^	32.3 ± 3.9 ^b^	32.4 ± 4.2 ^b^	32.3 ± 2.7 ^b^	31.7 ± 3.8 ^b^	32.9 ± 6.2 ^b^
**Perimeter (µm)**	21.9 ± 1.8 ^a^	20.1 ± 1.9 ^b^	22.8 ± 1.8 ^a^	22.9 ± 1.9 ^a^	22.9 ± 1.0 ^a^	22.7 ± 1.6 ^a^	23.1 ± 2.6 ^a^
**Post-thawed sperm midpiece trait**							
**Width (µm)**	1.3 ± 0.5 ^a^	1.0 ± 0.3 ^a^	1.0 ± 0.4 ^a^	1.0 ± 0.3 ^a^	1.0 ± 0.2 ^a^	1.1 ± 0.4 ^a^	1.1 ± 0.5 ^a^
**Post-thawed sperm shape indices**							
**Ellipticity**	2.0 ± 0.2	1.9 ± 0.2	1.9 ± 0.2	1.9 ± 0.8	1.9 ± 0.2	1.9 ± 0.3	2.0 ± 0.6
**Elongation**	0.3 ± 0.0	0.3 ± 0.0	0.3 ± 0.0	0.3 ± 0.1	0.3 ± 0.0	0.3 ± 0.0	0.3 ± 0.1
**Regularity**	0.8 ± 0.1	0.9 ± 0.1	0.9 ± 0.0	0.9 ± 0.1	0.9 ± 0.0	0.9 ± 0.0	0.9 ± 0.0
**Roughness**	1.2 ± 0.1	0.8 ± 0.1	0.8 ± 0.0	0.8 ± 0.1	0.8 ± 0.0	0.8 ± 0.0	0.8 ± 0.1

^a–c^ Values with different superscripts differ significantly in the same row (*p* < 0.05).

## Data Availability

The data is available from the corresponding author on request.
